# The loss of plant functional groups increased arthropod diversity in an alpine meadow on the Tibetan Plateau

**DOI:** 10.3389/fpls.2024.1305768

**Published:** 2024-02-16

**Authors:** Ningna Lu, Hainian Yang, Xianhui Zhou, Yun Tan, Wei Cai, Qin Jiang, Ying Lu, Yangyang Chen, Haocheng He, Sheng Wang

**Affiliations:** ^1^ College of Life Science, Northwest Normal University, Lanzhou, China; ^2^ State Key Laboratory of Herbage Improvement and Grassland Agro-ecosystems, College of Ecology, Lanzhou University, Lanzhou, China

**Keywords:** arthropods, species diversity, arthropod diversity, alpine meadow, functional groups

## Abstract

Plant species loss, driven by global changes and human activities, can have cascading effects on other trophic levels, such as arthropods, and alter the multitrophic structure of ecosystems. While the relationship between plant diversity and arthropod communities has been well-documented, few studies have explored the effects of species composition variation or plant functional groups. In this study, we conducted a long-term plant removal experiment to investigate the impact of plant functional group loss (specifically targeting tall grasses and sedges, as well as tall or short forbs) on arthropod diversity and their functional groups. Our findings revealed that the removal of plant functional groups resulted in increased arthropod richness, abundance and the exponential of Shannon entropy, contrary to the commonly observed positive correlation between plant diversity and consumer diversity. Furthermore, the removal of different plant groups had varying impacts on arthropod trophic levels. The removal of forbs had a more pronounced impact on herbivores compared to graminoids, but this impact did not consistently cascade to higher-trophic arthropods. Notably, the removal of short forbs had a more significant impact on predators, as evidenced by the increased richness, abundance, the exponential of Shannon entropy, inverse Simpson index and inverse Berger-Parker index of carnivores and abundance of omnivores, likely attributable to distinct underlying mechanisms. Our results highlight the importance of plant species identity in shaping arthropod communities in alpine grasslands. This study emphasizes the crucial role of high plant species diversity in controlling arthropods in natural grasslands, particularly in the context of plant diversity loss caused by global changes and human activities.

## 1 Introduction

Global changes (Butchart et al., 2010; Griggs et al., 2013; Maes et al., 2016) and human activities have resulted in a decline in biodiversity (Dirzo et al., 2014; Vogel, 2017; Leather, 2018), which in turn affects ecosystem functioning and services (Loreau and Hector, 2001; Cardinale et al., 2012; Isbell et al., 2017). For instance, experimental warming and livestock grazing have been shown to significantly alter plant species composition and net primary production in alpine grasslands on the Tibetan Plateau, particularly the relative abundance of grasses, sedges, and herbs (Klein et al., 2007; Wang et al., 2012; Liu et al., 2018). However, the impact of plant species loss on other trophic levels, such as consumer diversity and multitrophic structure, has rarely been investigated in natural grasslands (Hooper et al., 2005; Duffy et al., 2007; Haddad et al., 2009; Scherber et al., 2010).

Arthropods play a vital role in grassland ecosystems and their abundance and community structure are greatly influenced by primary producers (Wilson, 1987; Siemann et al., 1998; Uchida & Ushimaru, 2014). A diverse plant community provides a wider range of resources and higher productivity, thereby supporting a greater variety of consumers (Strong, 1984; Srivastava & Lawton, 1998). For example, numerous studies have shown a positive relationship between plant diversity and herbivore diversity and abundance (Haddad et al., 2009; Ebeling et al., 2018). This relationship is attributed to the increased availability of ecological niches and dietary diversity for herbivores in diverse plant communities. As a result, the richness of carnivores is also promoted due to the higher diversity and abundance of herbivores (Siemann et al., 1998; Haddad et al., 2001; Gamfeldt et al., 2005; Dassou & Tixier, 2016; Jacquot et al., 2019). This is consistent with the resource heterogeneity hypothesis (Hutchinson, 1959; Lewinsohn & Roslin, 2008; Moreira et al., 2016), as a greater diversity of herbivores provides more specialized prey for certain carnivores, leading to an increase in their richness. Additionally, diverse plant communities can provide more structurally diverse habitats (Strong, 1984), which can support higher abundances of predators and limit herbivore populations (the Enemies Hypothesis, Root, 1973). While many manipulating biodiversity experiments have found a positive correlation between plant diversity and consumer diversity (herbivores and carnivores) (Koricheva et al., 2000; Haddad et al., 2001; Wimp et al., 2004; Crutsinger et al., 2006; Johnson et al., 2006; Haddad et al., 2009; Scherber et al., 2010; Borer et al., 2012; Ebeling et al., 2018), there have been a few observational and experimental studies that reported no impact of plant diversity on consumer diversity (Currie, 1991; Wright & Samways, 1998; Hawkins & Porter, 2003; Jetz et al., 2009).

While the relationships between plant diversity and arthropod communities have been well-documented, few studies have specifically investigated the impact of variation in plant species composition or functional groups on these communities (Schaffers et al., 2008). It is important to consider the influence of plant composition and functional groups as they form the basis of arthropod food webs by providing resources such as foliage, nectar, and pollen (Bronstein et al., 2006; Gish et al., 2015). Many phytophagous insects rely on specific plant species or genera for their survival (Bernays & Graham, 1988; Forister et al., 2015). Therefore, changes in plant species composition or functional groups can have a significant impact on herbivores and other higher trophic level arthropods (Symstad et al., 2000; Schaffers et al., 2008; Tobisch et al., 2023).

To address this knowledge gap, this study utilizes a long-term experiment in an alpine meadow on the Tibetan Plateau, where different plant functional groups have been systematically removed since 2012 (Zhou, 2019). Specifically, plant species from different height categories, including tall grass and sedge species, tall forb species, and short forb species, were targeted for removal. The experiment focuses on the impacts of plant diversity loss on arthropod diversity and their functional groups. This approach reflects realistic scenarios observed in ecosystems affected by overgrazing (Mu et al., 2016; Wang and Wesche, 2016; Niu et al., 2016a; Abdalla et al., 2018), human activities (such as grazing exclusion and fertilizer addition), and climate change (such as experimental warming) (Klein et al., 2007; Chen et al., 2013; Niu et al., 2014; Zhang et al., 2015), which can lead to changes in plant species abundance and composition.

The study aims to address the following questions: 1) Does the removal of plant species result in a decrease in arthropod abundance and diversity? 2) How does the removal of different plant functional groups affect arthropod communities across various trophic levels, such as herbivores, carnivores, and omnivores? Based on the well-established positive relationship between plant diversity and arthropod diversity, we predict that the overall diversity and abundance of arthropods will decrease in response to the removal of any of the three targeted plant groups. Furthermore, we expect that different plant groups will have varying impacts on arthropod trophic levels, which can be attributed to different mechanisms.

## 2 Materials and methods

### 2.1 Study site

The experiment was conducted in the Gansu Gannan Grassland Ecosystem National Observation and Research Station (35°58’N, 101°53’E) located in Maqu County of Gansu Province. The site experiences more than 2580 hours of annual sunshine, with no absolute frost-free period throughout the year and an annual average frost period of no less than 270 days. The average annual temperature is 1.20°C, with the highest temperature during the growing season ranging from 23.6-28.9°C. The average annual precipitation is 620-780mm, mainly occurring from June to September.

The vegetation at the site is typical alpine meadow on the eastern Tibetan Plateau, with *Kobresia setschwanensis* as the dominant species and other abundant grass and forb species such as *Elymus nutans*, *Koeleria cristata*, *Anemone obtusiloba*, and *Anemone rivularis* (**Supplementary Figure S1**). These species account for more than 80% of the total aboveground biomass of the quadrat plant community (Zhou, 2019).

### 2.2 Species removal experiment

The plant species removal experiment was established in a fenced alpine meadow in 2012, with no grazing from April to November (Zhou, 2019; Zhou et al., 2019). The experiment included five replicates for each treatment, with a total of 20 1.5m×1.5m plots (**Supplementary Figure S2**). This design was chosen to balance longer periods of field work with a large labor force, due to the relatively low β diversity of the plant community and soil heterogeneity (Niu et al., 2016b). Following a typical random blocks experimental design, we established 5 blocks as repetitions for plant removal. The removal was carried out once a year, in the beginning of the growing season in May since 2012. The target plant species (**Supplementary Table S1**) are removed with hand scissors, at the soil surface leaving the rest of the vegetation undisturbed. It is important to note that when removing the target plant species, we made efforts to remove the roots without damaging neighboring non-target individuals. Considering the actual loss of plant species diversity due to land use and climate change, we implemented four treatments: Control without removal (CK), removal of tall forb species (B: Re_tall_Forbs), removal of short forb species (C: Re_short_Forbs), and removal of tall grass and sedge species (D: Re_tall_Grasses_Sedges).

Numerous studies have demonstrated that height is a crucial trait in determining species fitness (Westoby et al., 2002; Díaz et al., 2016; Thomson et al., 2018; Du et al., 2022). Fertilization and removal experiments conducted on the Qinghai-Tibet Plateau have also shown that height differences play a dominant role in species fitness differences, species coexistence, and productivity in alpine grassland plant communities (Liu et al., 2016). Given the large number of forb species, we categorized them into tall (above 30cm) and short (below 15cm) categories to balance the number of species and biomass in the experimental site (**Supplementary Table S1**). The number of species removed varied by among treatments (Re_tall_Forbs: 19 species; Re_short_Forbs: 19 species; Re_tall_Grasses_Sedges: 12 species). Importantly, this approach ensures that the loss of species diversity and biomass is comparable between treatments.

### 2.3 Measurement of plant community

Three surveys were conducted to measure the plant community in each removal plot from late July to late August 2021. Within a 50 cm×50 cm quadrat, we measured and counted the plant species, as well as the height and abundance of each species in the plot. During the third plant quadrat survey, we collected plant material by cutting all plants in each plot at the soil surface, and then oven-dried the samples at 75 °C for 48 hours and measured their dry biomass (g m^-2^).

### 2.4 Arthropod sampling

For arthropod sampling, we used a trap method to collect surface arthropods three times in early July, early August, and late August 2021. It needs to note that no pre-treatment data of arthropods are available. Prior to sampling, we checked the local weather forecast for the next 48 hours and chose clear weather. To minimize damage to the plant community, we laid 20 trap bottles for each sampling site according to the plant growth of each sample site. Each trap bottle had a top diameter of 5.7 cm, a bottom diameter of 5.7 cm, a height of 7.1 cm, and a volume of approximately 200 ml. We poured 50-70 ml of 20% alcohol solution and 10-20 ml of white vinegar into each trap bottle, buried it in the ground and kept the mouth level with the surface. After 48 hours, we retrieved the trap bottles and separated the collected surface arthropods, which were then stored in 75% alcohol liquid. In the laboratory, we observed and identified the arthropods at least to the family level, or where possible, to the genus and species level (Ward et al., 2001). We recorded the species abundance for each quadrat and classified all arthropods into different functional groups based on taxonomy, life history characteristics, and feeding relationships (**Supplementary Table S2**).

### 2.5 Statistical analysis

Due to the random block design and repeated measurements, the experimental data are not completely independent. Therefore, to examine the overall impacts of plant species loss on arthropods, we conducted statistical analyses using linear mixed-effects models (LMMs). We calculated the effect size and its corresponding 95% confidence interval (CI) to quantify the treatment effect of plant species loss on arthropods. The regression coefficient in the LMMs represents the direction and magnitude of the treatment effect (Wu et al., 2022). Before analysis, we performed a natural logarithmic transformation on the response variable data to improve normality.

To visualize the differences in arthropod species composition under different treatments, we used non-metric multidimensional scaling (NMDS) and performed Permutational multivariate analysis of variance (PERMANOVA). The “metaMDS” function in the “vegan” package was used for NMDS, and a distance matrix was created based on arthropod abundance data using Bray-Curtis coefficients. Bray-Curtis distances were then used for NMDS ordering. All analyses were permuted 999 times (Minchin, 1987; Legendre and Legendre, 1998).

Furthermore, to investigate the effects of species loss on arthropod species diversity, we estimated five measures: (a) arthropod abundance, which refers to the number of individual arthropods; (b) arthropod richness, which refers to the number of arthropod species; (c) the exponential of Shannon entropy (Chao et al., 2014), which is often sensitive to the presence of rare species (Nagendra, 2002); (d) the inverse Simpson index, which emphasizes the dominant members (Nagendra, 2002), can be interpreted as the effective number of dominant species in the assemblage; (e) inverse Berger-Parker index, which is a preponderance measure that simply represents the proportional abundance of the most abundant species (Magurran, 2004). The four diversity indices can give an emphasis respectively on total species (species richness), rare species (Shannon diversity), dominant species (Simpson diversity) and the top dominant species (Berger-Parker) (**Supplementary Table S6**).

Based on correlations between our observations and data formats, as well as parameters for more accurate estimates of complex data structures. We used LMMs to examine the impacts of plant species diversity on arthropod and functional groups diversity (i.e. abundance, richness, the exponential of Shannon entropy, inverse Simpson index and inverse Berger-Parker index). Overall, arthropod diversity as a response variable and plant species diversity as a predictor explained the relationship between arthropods and herbivores and plant diversity. At the same time, we also analyzed the correlation between the diversity of different functional groups of arthropods, and set the corresponding prediction and response variables according to their significant difference changes under different treatments and the cascade relationship between different trophic levels. The all LMMs included the block as an independent random intercept effect.

All data analysis was conducted in R version 4.3.1 (http://www.r-project.org.version4.3.1). The “vegan” package was used for NMDS, and the “lme4” package was used for LMMs (Bates et al., 2015). We calculated the marginal and conditional R^2^-values of the models using the “glmm.hp” package (Lai et al., 2022). We use the “iNEXT” package to calculate R/E curves and sample completeness curves based on sample size. The results showed that the sampling effort was adequate, respectively covering (98.0%, 98.2%, 98.9%, 98.7%) of species richness in the communities (**Supplementary Figure S3**).

## 3 Results

### 3.1 Effects of plant functional groups removal on arthropod community

In the removing experiments, 2659 arthropods were totally sampled. Herbivores, carnivores, and omnivores accounted for 36.5%, 11.25%, and 52.25% of all arthropods in the control, 25.36%, 2.37%, and 72.27% in the treatment of removing tall grasses and sedges, 28.55%, 15.82%, and 55.63% in the treatment of removing tall forbs, and 29.94%, 11.75%, and 58.31% in the treatment of removing short forbs.

**Figure 1:**
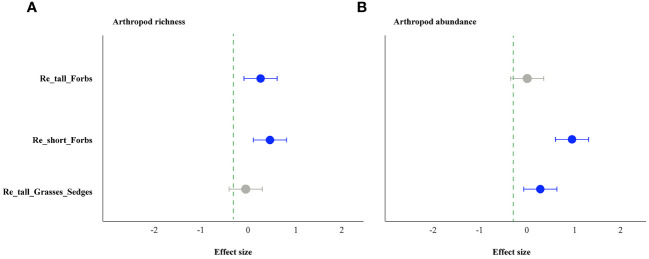
The effects of removing plant functional groups on arthropod richness **(A)** and abundance **(B)** by linear mixed-effects models (LMMs). The estimated values of the LMMs represent the positive (blue) or negative (red) effects of different species removals. The uncertainty associated with each treatment is depicted by the median values (above the bars) and a 95% credibility interval. A significant effect is indicated when the 95% confidence interval error line does not overlap with 0. The treatments include the removal of tall forb species (Re_tall_Forbs), short forb species (Re_short_Forbs), and tall grasses and sedges (Re_tall_Grasses_Sedges).

**Figure 2:**
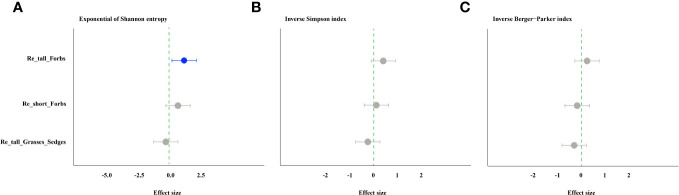
The effects of removing plant functional groups on the exponential of Shannon entropy **(A)**, inverse Simpson index **(B)** and inverse Berger-Parker index of arthropod **(C)** by linear mixed-effects models (LMMs). The estimated values of the LMMs represent the positive (blue) or negative (red) effects of different species removals. The comments in the figure correspond to those shown in **Figure 1**.

The forbs removal (including tall and short) significantly increased arthropod species richness, while removing tall grasses and sedges did not (**Figure 1A**). The abundance of arthropods increased significantly in the treatments of removing tall grasses and sedges and short forbs, but not in the treatment of removing tall forbs. Specifically, the former showed a 101% increase, and the latter showed a 150% increase (**Figure 1B**). For arthropods in the remove tall forbs treatment, the exponential of Shannon entropy was increased significantly, but did not change in the treatments of removing short forbs and tall grasses and sedges (**Figure 2A**). In the treatments involving the removal of tall grasses and sedges and forbs, there was no change observed in the inverse Simpson index and inverse Berger-Parker index of arthropods (**Figures 2B, C**).

**Figure 3:**
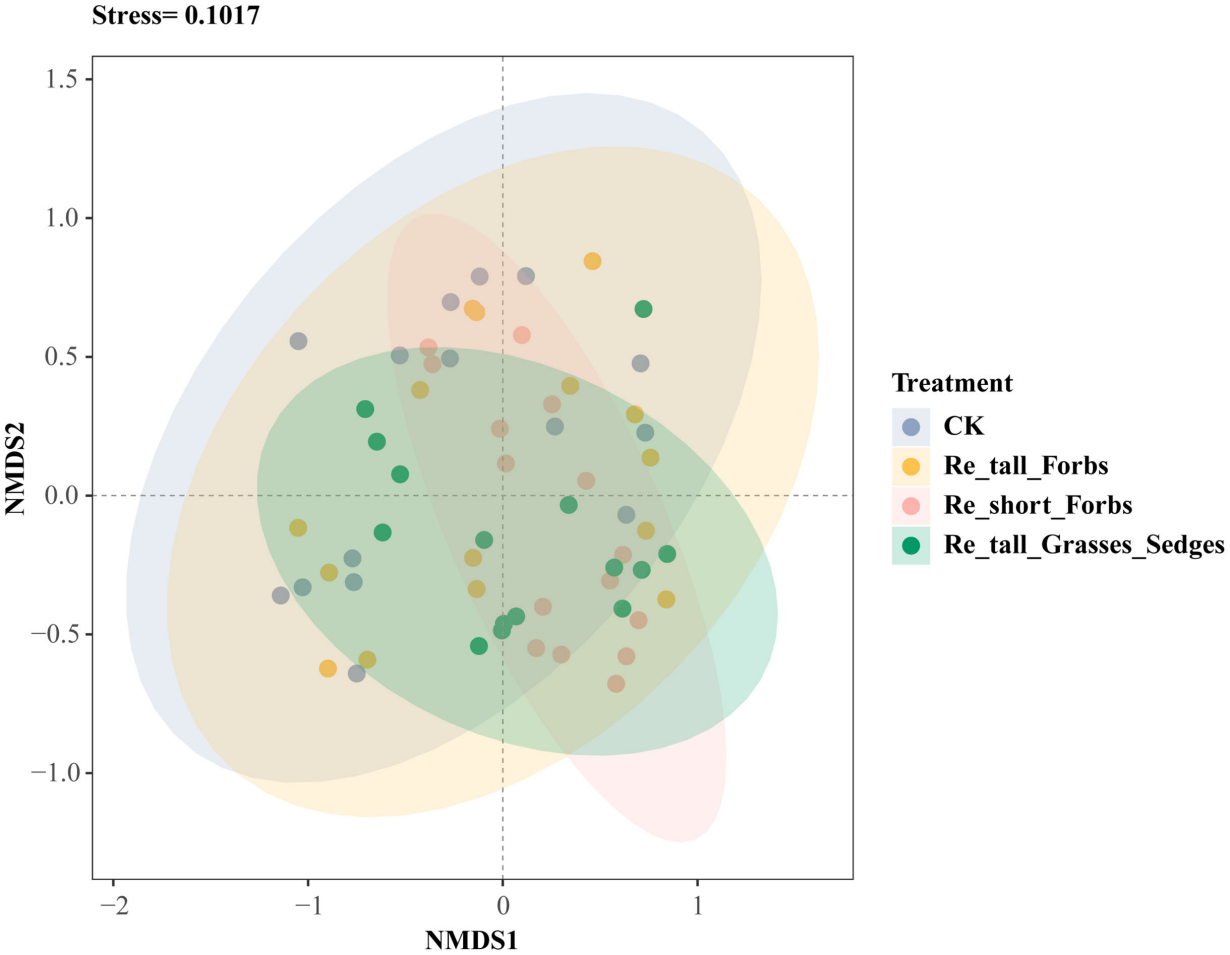
The differences in arthropod species composition between the treatments and the control shown by nonmetric multidimensional scaling analysis (NMDS).

Overall, there was no significant correlation between arthropod richness, the exponential of Shannon entropy, inverse Simpson index and inverse Berger-Parker index and plant diversity (**Supplementary Figure S8**), while higher plant diversity significantly reduced arthropod abundance (**Supplementary Figure S7**). Furthermore, compared to the control, both removing short forbs and tall grasses and sedges significantly altered the species composition of the arthropod community (**Figure 3**; **Supplementary Table S3**).

### 3.2 Effects of plant functional groups removal on the trophic groups of arthropods

**Figure 4:**
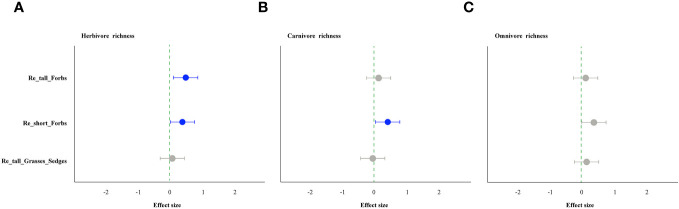
The effect of plant species removal on the richness of arthropod functional groups, as determined by linear mixed-effects models (LMMs). Herbivores **(A)**, carnivores **(B)**, and omnivores **(C)**. The comments in the figure correspond to those shown in **Figure 1**.

**Figure 5:**
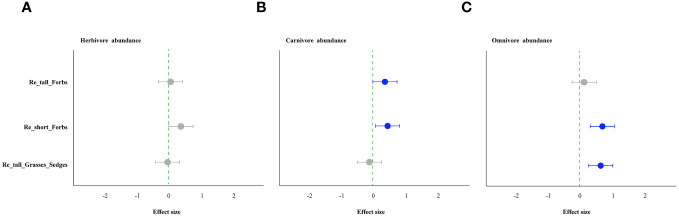
The effect of plant species removal on the abundance of arthropod functional groups by linear mixed-effects models (LMMs). Herbivore **(A)**, carnivore **(B)**, and omnivore **(C)**. The comments in the figure correspond to those shown in **Figure 1**.

**Figure 6:**
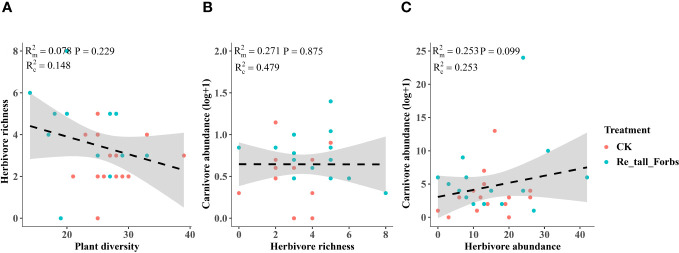
The relationships between plant diversity and the diversity of arthropod functional groups in the removal of tall forbs and the control. Plant diversity and herbivore richness **(A)**, herbivore richness and carnivore abundance **(B)**, the relationship between herbivore abundance and carnivore abundance **(C)**. The figure includes the R^2^ value and P value of the regression model, along with a 95% confidence interval.

As comparing the treatments to the control, the removal of tall and short forbs had a greater effect on the various trophic arthropods compared to the removal of tall grasses and sedges. Specifically, the removal of tall forbs significantly increased herbivore richness and carnivore abundance (**Figures 4A**, **5B**). However, herbivore abundance did not change in the treatment of removing forbs and tall grasses and sedges (**Figure 5A**). The increase in herbivore richness was a direct effect of the treatment and was not influenced by plant diversity (**Figure 6A**). Similarly, the variation in carnivore abundance was a direct result of the treatment, as there was no significant relationship between carnivore abundance and herbivore richness or abundance (**Figures 6B, C**).

**Figure 7:**
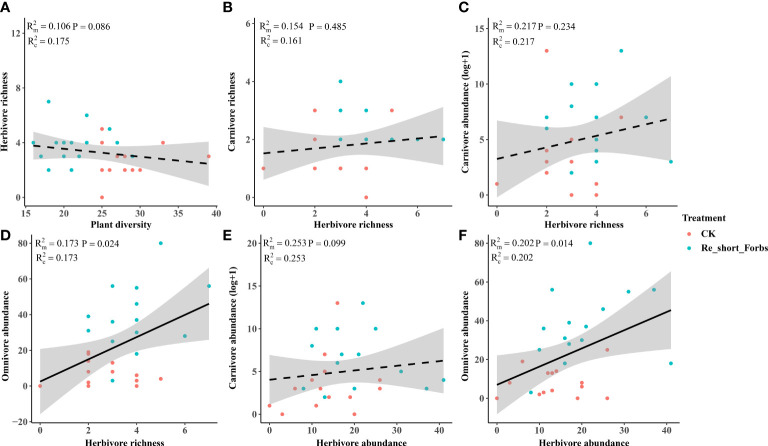
The relationships between plant diversity and arthropod trophic groups in the removal of short forbs and the control. Plant diversity and herbivore richness **(A)**, herbivore richness and carnivore richness **(B)**, herbivore richness and carnivore abundance **(C)**, herbivore richness and omnivore abundance **(D)**, herbivore abundance and carnivore abundance **(E)**, herbivore abundance and omnivore abundance **(F)**. The figure includes the R^2^ value and P value of the regression model, along with a 95% confidence interval.

The removal of short forbs significantly increased the richness (**Figure 4**), the exponential of Shannon entropy (**Supplementary Figure S4**), inverse Simpson index (**Supplementary Figure S5**) and inverse Berger-Parker index (**Supplementary Figure S6**) of herbivores and carnivores, as well as the abundance of carnivores (**Figure 5B**) and omnivores (**Figure 5C**). The higher herbivore richness observed was a direct consequence of the treatment, as there was no correlation between herbivore richness and plant diversity (**Figure 7A**). The increased richness and abundance of carnivores were also direct outcomes of the treatment, as they were not related to herbivore richness and abundance (**Figures 7B, C, E**). However, the higher abundance of omnivores in the treatment was driven by the increased herbivore richness and abundance, as indicated by their significant correlations (**Figures 7D, F**). For carnivores in the treatment, the higher the exponential of Shannon entropy was driven by the increased herbivore richness, due to their significant correlation (**Supplementary Figure S9**). However, the exponential of Shannon entropy of herbivore, inverse Simpson index and inverse Berger-Parker index of herbivores and carnivores were direct outcomes of the treatment, as they were not related to plant diversity and herbivore richness (**Supplementary Figure S9**).

**Figure 8:**
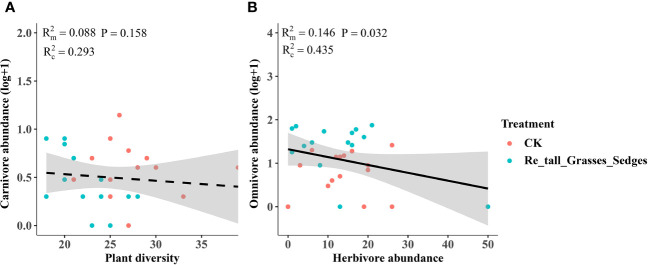
The relationship between plant diversity and the abundance of arthropod functional groups in the removal of tall grasses and sedges and the control. Plant diversity and carnivore abundance **(A)**, herbivore abundance and omnivore abundance **(B)**. The figure includes the R2 value and P value of the regression model, along with a 95% confidence interval.

In contrast, the removal of tall grasses and sedges only significantly increased the abundance of omnivores (**Figure 5C**). Furthermore, there was a negative correlation between omnivore abundance and herbivore abundance (**Figure 8B**).

## 4 Discussion

A diverse plant community can provide a wide range of resources and higher productivity, which in turn supports a greater diversity of consumers (Strong, 1984; Srivastava & Lawton, 1998). Numerous studies have reported a positive correlation between plant diversity and consumer diversity, including herbivores and carnivores, in manipulating biodiversity experiments (Koricheva et al., 2000; Haddad et al., 2001; Wimp et al., 2004; Crutsinger et al., 2006; Johnson et al., 2006; Haddad et al., 2009; Scherber et al., 2010; Borer et al., 2012; Ebeling et al., 2018). However, our long-term removal experiment yielded unexpected results, as we found that the loss of plant species increased both the richness and abundance of arthropods, especially the number of rare species (exponential of Shannon entropy) of arthropods, contrary to our initial prediction. Additionally, we observed different impacts of plant functional groups on arthropods. Specifically, the removal of forbs (including tall and short) significantly increased arthropod species richness, even higher the exponential of Shannon entropy after removing tall forbs, while the loss of tall grasses and sedges, or short forbs, significantly increased arthropod abundance. These removal treatments also led to changes in the species composition of arthropod communities, despite the absence of an overall correlation between plant diversity and arthropod richness. These findings indicate that high plant species diversity in natural grasslands on the Tibetan Plateau plays a crucial role in controlling the diversity and abundance of arthropods, as well as maintaining interactions within food webs (Barnes and Scherber, 2020).

Furthermore, our study revealed that the removal of different plant groups had varying impacts on arthropods across trophic levels, potentially due to different underlying mechanisms. Diverse plant functional groups can provide distinct food resources for arthropods, thus influencing their composition and diversity. For instance, legumes (e.g. *Oxytropis kansuensis*, *Oxytropis ochrocephala*, *Tibetia himalaica*), which contain high levels of nitrogen, can serve as a high-quality resource for certain herbivores (Kareiva, 1984). In contrast, gramineous plants are known for their low ammonia and hardness levels (Symstad et al., 2000), resulting in lower quality food resources for arthropods (Pinder and Kroh, 1987). Our study found that the removal of forbs had a greater impact on arthropods across different trophic levels compared to the removal of tall grasses and sedges, with differences also observed between tall and short forbs. The loss of both tall and short forbs, rather than tall graminoids, significantly increased herbivore richness (**Figure 4A**). Specifically, the loss of short forbs significantly increased the diversity of rare and dominant herbivores (**Supplementary Figures S4**–**S6**). This contrasts with a previous study that showed some orders of herbivores positively responded to the presence of forbs (Symstad et al., 2000). The observed impact of treatment may not be a result of indirect plant diversity loss, as herbivore richness was not related to plant diversity (**Figures 6A**, **7A**). Instead, this may correspond to the resource concentration hypothesis (Root, 1973), which suggests that specialist herbivores are attracted to their host plants when other forbs are removed, leading to an increase in herbivore richness. This hypothesis is based on the idea that high plant diversity will increase nutrient heterogeneity within the plant community, and high-quality resources will be diluted, leading to a reduction in arthropod diversity (Otway et al., 2005). Additionally, nitrogen levels within plant species were found to be reduced in plant communities with high plant species richness (van Ruijven and Berendse, 2005; Borer et al., 2015), suggesting that plant species nutrition may be lower in communities with high plant diversity. Surprisingly, the removal of tall grasses and sedges did not have a significant impact on herbivores, which is inconsistent with several studies that have shown the presence of grasses significantly decreases herbivory rates or some orders of herbivores (Symstad et al., 2000; Loranger et al., 2014).

Although removing forbs increased herbivore, we did not observe consistent cascading impacts on higher-trophic predators. In the treatment where tall forbs were removed, the increased abundance of carnivores may be a direct result of the treatment, as there was no significant relationship between carnivore abundance and herbivore richness and abundance (**Figures 6B, C**). On the other hand, the removal of short forbs had a significant impact on high-trophic arthropods, with the richness, abundance, diversity of rare and dominant carnivores and abundance of omnivores being significantly increased by different mechanisms. The increased carnivore abundance may also be a direct result of the treatment, whereas the higher omnivore abundance observed in the treatment was likely a trophic cascading impact caused by the increased herbivore richness and abundance (**Figures 7D, F**). For carnivores (i.e., rare species) in the treatment, the higher the exponential of Shannon entropy observed was also likely caused by trophic cascading effect as its significant correlation with herbivore richness (**Supplementary Figure S9**). Similar results have been observed in other studies, where predator abundance and species richness decreased with increasing tree species richness (Schuldt and Assmann, 2011). In our study, the direct impact of removing forbs on carnivores abundance and dominate species diversity of carnivores may be independent of trophic mechanisms mediated by the herbivore community. Instead, changes in abiotic conditions produced by removing forbs in the plant community could be more relevant to predator communities (van Schalkwyk et al., 2019; Tobisch et al., 2023). The removal of forbs, especially short forbs, could decrease structural complexity and increase activity space under the vegetation due to their high cover. This variation in vegetation structure or local habitat conditions may be more important for predators (Brose, 2003; Schaffers et al., 2008; Tobisch et al., 2023), such as enhancing activity and thus predation rate.

In contrast, the removal of tall grasses and sedges significantly increased omnivore abundance rather than carnivore abundance (**Figures 5C**, **8A**). The higher omnivore density observed after removing grasses and sedges may also be a result of changes in microhabitats, in contrast to the treatment of removing short forbs where higher omnivore density was likely caused by the trophic impact of increased herbivores. The increased omnivore abundance in the treatment of removing tall grasses and sedges may be responsible for the control of herbivores, as there was a negative correlation between omnivore abundance and herbivore abundance. This top-down impact may be an important reason why herbivore abundance did not change after removing graminoids. The differential impacts of the three plants groups’ removal suggest that plant species composition, specifically the identity of plant species in the community, is a more important determinant of arthropods in grasslands than plant species richness per se or the number of plant functional groups (Koricheva et al., 2000; Symstad et al., 2000; Loranger et al., 2014).

## 5 Conclusion

This study demonstrated that the loss of plant functional groups increased arthropod richness and abundance and the diversity of rare arthropod species, which contrasts with the well-reported positive correlation between plant diversity and consumer diversity in manipulating biodiversity experiments, but consistent with agricultural experiments using multi-species mixtures (e.g., reviewed in Andow, 1991). There were differences in the impacts of plant functional group loss, leading to changes in the species composition of arthropod communities. Furthermore, the removal of different plant groups had differential impacts on arthropod trophic levels. More evident impacts of removing forbs rather than graminoids on herbivores did not show consistent cascading impacts on higher-trophic carnivores, possibly due to different underlying mechanisms. Our results highlight the importance of plant species identity as a determinant of arthropods in alpine grasslands, surpassing the significance of plant species richness or functional groups. This study emphasizes the role of high plant species diversity in controlling arthropods in natural grasslands, particularly in the face of plant diversity loss caused by global changes and human activities.

## Data availability statement

The raw data supporting the conclusions of this article will be made available by the authors, without undue reservation.

## Ethics statement

Ethical approval was not required for the studies involving humans because this research was done jointly by the research team and members of other universities, all of whom are postgraduate students or above. The studies were conducted in accordance with the local legislation and institutional requirements. The participants provided their written informed consent to participate in this study. Ethical approval was not required for the study involving animals in accordance with the local legislation and institutional requirements because this study illustrates the relationship between plants and arthropods, and does not involve large animals and pets.

## Author contributions

NL: Conceptualization, Funding acquisition, Project administration, Resources, Supervision, Writing – review & editing. HY: Data curation, Formal analysis, Investigation, Methodology, Project administration, Software, Supervision, Visualization, Writing – original draft. XZ: Project administration, Supervision, Writing – review & editing. YT: Methodology, Supervision, Validation, Writing – original draft. WC: Investigation, Validation, Writing – original draft. QJ: Investigation, Validation, Writing – original draft. YL: Validation, Visualization, Writing – original draft. YC: Data curation, Validation, Writing – original draft. HH: Software, Validation, Writing – original draft. SW: Formal analysis, Investigation, Validation, Writing – original draft.
