# Supplementary material for: The loss of plant functional groups increased arthropod diversity in an alpine meadow on the Tibetan Plateau
Source: Front Plant Sci. 2024 Feb 16;15:1305768. doi: 10.3389/fpls.2024.1305768 (PMC10904612; doi:10.3389/fpls.2024.1305768)
Supplement: Supplementary file 1 [file DataSheet_1.docx]

Supplementary Material

# Supplementary Tables and Figures

## 1. Supplementary Tables

Table S1. List of excluded species

| Category | Species | Functional group | Height（cm） |
| --- | --- | --- | --- |
| Dicotyledon | *Anemone obtusiloba* | tall Forb | >30 |
| Dicotyledon | *Allium sikkimense* | tall Forb | >30 |
| Dicotyledon | *Anemone trullifolia var. linearis* | tall Forb | >30 |
| Dicotyledon | *Halenia elliptica* | tall Forb | >30 |
| Dicotyledon | *Delphinium kamaonense* | tall Forb | >30 |
| Dicotyledon | *Anemone rivularis* | tall Forb | >30 |
| Dicotyledon | *Pleurospermum uralense* | tall Forb | >30 |
| Dicotyledon | *Veronica eriogyne* | tall Forb | >30 |
| Dicotyledon | *Thermopsis lanceolata* | tall Forb | >30 |
| Dicotyledon | *Gentianopsis barbata* | tall Forb | >30 |
| Dicotyledon | *Rumex acetosa* | tall Forb | >30 |
| Dicotyledon | *Veronica vandellioides* | tall Forb | >30 |
| Dicotyledon | *Aster tataricus* | tall Forb | >30 |
| Dicotyledon | *Artemisia sieversiana* | tall Forb | >30 |
| Dicotyledon | *Artemisia frigida* | tall Forb | >30 |
| Dicotyledon | *Artemisia mongolica* | tall Forb | >30 |
| Dicotyledon | *Ligularia sibirica* | tall Forb | >30 |
| Dicotyledon | *Bupleurum hamiltonii* | tall Forb | >30 |
| Dicotyledon | *Polygonum viviparum* | tall Forb | >30 |
| Dicotyledon | *Gueldenstaedtia verna* | short Forb | 0-15 |
| Dicotyledon | *Taraxacum mongolicum* | short Forb | 0-15 |
| Dicotyledon | *Saussurea pachyneura* | short Forb | 0-15 |
| Dicotyledon | *Lamiophlomis rotata* | short Forb | 0-15 |
| Dicotyledon | *Euphrasia pectinata* | short Forb | 0-15 |
| Dicotyledon | *Oxytropis ochrocephala* | short Forb | 0-15 |
| Dicotyledon | *Saussurea stella* | short Forb | 0-15 |
| Dicotyledon | *Plantago asiatica* | short Forb | 0-15 |
| Dicotyledon | *Potentilla anserina* | short Forb | 0-15 |
| Dicotyledon | *Leontopodium leontopodioides* | short Forb | 0-15 |
| Dicotyledon | *Lancea tibetica* | short Forb | 0-15 |
| Dicotyledon | *Saussurea hieracioides* | short Forb | 0-15 |
| Dicotyledon | *Trigonotis peduncularis* | short Forb | 0-15 |
| Dicotyledon | *Nardostachys jatamansi* | short Forb | 0-15 |
| Dicotyledon | *Tibetia himalaica* | short Forb | 0-15 |
| Dicotyledon | *Medicago ruthenica* | short Forb | 0-15 |
| Dicotyledon | *Herminium monorchis* | short Forb | 0-15 |
| Dicotyledon | *Gentiana macrophylla* | short Forb | 0-15 |
| Dicotyledon | *Galium spurium* | short Forb | 0-15 |
| monocotyledon | *Elymus nutans* | tall Grass | >30 |
| monocotyledon | *Deschampsia cespitosa* | tall Grass | >30 |
| \| monocotyledon \| *Agrostis hugoniana* \| tall Grass \| >30 \| \| --- \| --- \| --- \| --- \|   Table S1. List of excluded species | | | |
| Category | Species | Functional group | Height（cm） |
| monocotyledon | *Agropyron cristatum* | tall Grass | >30 |
| monocotyledon | *Leymus secalinus* | tall Grass | >30 |
| monocotyledon | *Koeleria macrantha* | tall Grass | >30 |
| monocotyledon | *Stipa aliena* | tall Grass | >30 |
| monocotyledon | *Poa annua* | tall Grass | >30 |
| monocotyledon | *Festuca sinensis* | tall Grass | >30 |
| monocotyledon | *Kobresia pygmaea* | Sedge | >0 |
| monocotyledon | *Carex dahurica* | Sedge | >0 |
| monocotyledon | *Kobresia capillifolia* | Sedge | >0 |

Table S2. Community composition and relative abundance of ground arthropods/%

| Order | Family/Species | Functional  Group | Treatment | | | |  |
| --- | --- | --- | --- | --- | --- | --- | --- |
|  |  |  | CK | Re_tall_Forbs | Re_short_Forbs | Re_tall_Grasses_Sedges | |
| Hemiptera |  |  | 9.75 | 11.58 | 10.51 | 7.23 | |
|  | Cicadellidae | He | 6.00 | 4.40 | 2.60 | 2.37 | |
|  | Cercopidae | He | 2.50 | 2.45 | 3.95 | 0.66 | |
|  | Pentatomidae | He | 1.25 | 1.14 | 1.81 | 0.53 | |
|  | Lygaeidae | He |  | 0.16 |  | 0.13 | |
|  | Miridae | He |  | 0.16 | 0.11 |  | |
|  | Aphididae | He |  | 2.94 | 2.03 | 3.55 | |
|  | Psyllidae | He |  | 0.33 |  |  | |
| Lepidoptera |  |  | 1.50 | 1.79 | 1.13 | 1.05 | |
|  | *Lycaeides argyrognomon Bergstrasser* | He |  |  | 0.11 |  | |
|  | *Phassus excrescens* | He | 0.25 | 0.16 |  |  | |
|  | Noctuidae | He | 0.50 | 0.49 | 0.68 | 0.92 | |
|  | Noctuidae larvae | He | 0.50 | 0.49 |  |  | |
|  | Pyralidae | He | 0.25 |  |  |  | |
|  | Lasiocampidae | He |  |  |  | 0.13 | |
|  | *Aphantopus hyperanthus* | He |  | 0.49 | 0.23 |  | |
|  | Lasiocampa larvae | He |  | 0.16 | 0.11 |  | |
| Hymenoptera |  |  | 50.75 | 55.95 | 59.77 | 71.22 | |
|  | Formica | Om | 34.50 | 40.46 | 56.16 | 70.17 | |
|  | Syrphidae | Om | 2.25 | 1.14 |  | 0.39 | |
|  | *Pachyprotasis citrinipictus* | He |  | 0.16 | 1.02 |  | |
|  | *Monomorium pharaonis* | Om | 12.25 | 10.60 |  |  | |
|  | Pteromalidae | He | 0.25 | 0.49 | 0.34 | 0.53 | |
|  | Ichneumonidae | Om | 0.50 | 0.16 | 0.45 |  | |
|  | Crematogaster | Om |  | 1.31 |  |  | |
|  | Thyreusdecorus | Om | 0.25 |  | 0.11 | 0.13 | |
|  | Cerapachys Smith | Ca | 0.75 | 1.63 | 1.69 |  | |
| Coleoptera |  |  | 1.50 | 5.22 | 5.42 | 0.79 | |
|  | Curculionidae | He |  | 0.16 | 0.68 | 0.26 | |
|  | Eumolpidae | He | 0.25 |  |  |  | |
|  | Altica Goeffroy | He |  | 0.16 |  |  | |
|  | Carabidae | Ca | 0.50 | 0.82 | 0.11 | 0.26 | |
|  | Asilidae | Ca |  | 3.59 | 3.39 | 0.13 | |
|  | Scarabaeidae | He |  |  | 0.23 |  | |

Table S2. Community composition and relative abundance of ground arthropods/%

| Order | Family/Species | Functional  Group | Treatment | | | |  |
| --- | --- | --- | --- | --- | --- | --- | --- |
|  |  |  | CK | Re_tall_Forbs | Re_short_Forbs | Re_tall_Grasses_Sedges | |
|  | Staphylinidae | Ca | 0.50 | 0.33 | 0.68 |  | |
|  | Tenebrionidae | Ca | 0.25 |  |  | 0.13 | |
|  | Altica Goeffroy larvae | He |  |  | 0.23 |  | |
|  | Chrysomelidae | He |  |  | 0.11 |  | |
|  | Chrysomelidae larvae | He |  | 0.16 |  |  | |
| Diptera |  |  | 27.25 | 15.99 | 17.29 | 17.74 | |
|  | Tipulidae | He | 16.25 | 8.65 | 6.21 | 12.35 | |
|  | Chloropidae | He | 6.75 | 2.77 | 4.63 | 1.18 | |
|  | Agromyzidae | He | 1.25 | 2.28 | 2.94 | 2.50 | |
|  | Opomyzidae | He | 0.25 | 0.33 | 1.92 | 0.13 | |
|  | *Chrysomyini* | Om |  |  |  | 0.13 | |
|  | *Asteiidae* | He | 0.25 |  |  |  | |
|  | Culicidae | Om | 0.50 | 0.16 | 1.13 | 0.66 | |
|  | *Atylotus miser* | Om |  | 0.33 |  |  | |
|  | Anthomyiidae | Om | 1.25 | 0.65 | 0.34 | 0.13 | |
|  | Tachinidae | Om | 0.75 | 0.82 | 0.11 | 0.66 | |
| Araneae |  |  | 9.25 | 9.46 | 5.76 | 1.84 | |
|  | Ctenida | Ca | 3.75 | 6.53 |  | 0.66 | |
|  | Uloboridae | Ca | 3.00 | 2.94 | 4.29 | 1.18 | |
|  | Pachygnatha | Ca |  |  | 0.23 |  | |
|  | Zora spinimana | Ca | 2.50 |  | 0.11 |  | |
|  | Thomisidae | Ca |  |  | 0.90 |  | |
|  | Theridiidae | Ca |  |  | 0.23 |  | |
| Orthoptera |  |  |  |  |  | 0.13 | |
|  | *Dnopherula sinensis* | He |  |  |  | 0.13 | |
| Scolopendridae |  |  |  |  | 0.11 |  | |
|  | *Centipede* | Ca |  |  | 0.11 |  | |
| Total number of groups | |  | 29 | 36 | 35 | 26 | |
| Total number of individuals | |  | 400 | 613 | 885 | 761 | |

Note: He: Herbivore group; Ca: Carnivore group; Om: Omnivore group

Table S3. Multivariate analysis (PERMANOVA) of variance was substituted to explore the differences in arthropod community composition between control and treatment. Means significant difference is indicated by bold font.

| *Group* | *distance* | *Df* | Mean squares | Variation(R^2^) | Pr (>F) |
| --- | --- | --- | --- | --- | --- |
| Re_tall_Forbs/CK | Bray-Curtis | ***1*** | 0.2791 | 0.0319 | 0.468 |
| Re_short_Forbs/CK | Bray-Curtis | ***1*** | 1.0433 | 0.1271 | **0.006** |
| Re_tall_Grasses_Sedges/CK | Bray-Curtis | ***1*** | 0.9769 | 0.1219 | **0.006** |

Table S4. Results of a linear mixing model was the removal treatment and plant diversity on richness of arthropods. Sample months and blocks are added to the model as random effects. *Estimates*, *t-values* and *P values* for each explanatory variable are presented in the table. *P<0.05* means significant difference, *P<0.01* means extremely significant difference, and is indicated by bold font, *P>0.05* means no significant difference.

|  | Re_tall_Forbs | | | | Re_short_Forbs | | | | Re_tall_Grasses_Sedges | | | |
| --- | --- | --- | --- | --- | --- | --- | --- | --- | --- | --- | --- | --- |
| Explanatory variables | *Estimate* | *Std.error* | *t* | *p* | *Estimate* | *Std.error* | *t* | *p* | *Estimate* | *Std.error* | *t* | *p* |
| (Intercept) | 11.763 | 3.143 | 3.742 | 0.001 | 10.754 | 3.621 | 2.970 | 0.006 | 12.202 | 3.421 | 3.567 | 0.001 |
| Treatment | 0.888 | 3.836 | 0.232 | 0.819 | -1.781 | 4.869 | -0.366 | 0.718 | -7.226 | 5.314 | -1.360 | 0.186 |
| Plant diversity | -0.118 | 0.111 | -1.059 | 0.300 | -0.081 | 0.131 | -0.620 | 0.540 | -0.134 | 0.123 | -1.090 | 0.286 |
| Treatment×plant diversity | 0.037 | 0.146 | 0.255 | 0.801 | 0.160 | 0.199 | 0.805 | 0.428 | 0.356 | 0.218 | 1.631 | 0.115 |

Table S5. Results of a linear mixing model was the removal treatment and plant diversity on abundance of arthropods. Sample months and blocks are added to the model as random effects. *Estimates*, *t-values* and *P values* for each explanatory variable are presented in the table. *P<0.05* means significant difference, *P<0.01* means extremely significant difference, and is indicated by bold font, *P>0.05* means no significant difference.

|  | Re_tall_Forbs | | | | Re_short_Forbs | | | | Re_tall_Grasses_Sedges | | | |
| --- | --- | --- | --- | --- | --- | --- | --- | --- | --- | --- | --- | --- |
| Explanatory variables | *Estimate* | *Std.error* | *t* | *p* | *Estimate* | *Std.error* | *t* | *p* | *Estimate* | *Std.error* | *t* | *p* |
| (Intercept) | 44.327 | 35.694 | 1.242 | 0.227 | 45.101 | 37.561 | 1.201 | 0.241 | 54.245 | 33.271 | 1.630 | 0.117 |
| Treatment | 24.158 | 42.824 | 0.564 | 0.578 | 11.850 | 50.507 | 0.235 | 0.816 | -52.298 | 46.621 | -1.122 | 0.274 |
| Plant diversity | -0.311 | 1.273 | -0.245 | 0.809 | -0.339 | 1.355 | -0.250 | 0.804 | -0.673 | 1.168 | -0.576 | 0.570 |
| Treatment×Plant diversity | -0.454 | 1.622 | -0.280 | 0.782 | 1.370 | 2.067 | 0.663 | 0.513 | 3.643 | 1.923 | 1.894 | 0.071 |

| Table S6. Four Hill numbers are listed in the table, emphasis respectively on total species (species richness), rare species (Shannon diversity), dominant species (Simpson diversity) and the top dominant species (Berger-Parker). | | | | | | |
| --- | --- | --- | --- | --- | --- | --- |
| Treatment | Time | Category | Richness | Exponential of Shannon entropy | Inverse Simpson index | Inverse Berger-Parker index |
| CK | 1 | Arthropods | 5 | 1.5345 | 4.3689 | 3.3333 |
| CK | 1 | Arthropods | 6 | 1.3166 | 3.0433 | 2.4091 |
| CK | 1 | Arthropods | 4 | 1.1268 | 2.7325 | 2.3125 |
| CK | 1 | Arthropods | 10 | 1.8424 | 4.7711 | 2.9375 |
| CK | 1 | Arthropods | 10 | 2.0842 | 6.6977 | 4.0000 |
| CK | 2 | Arthropods | 7 | 1.2255 | 2.2092 | 1.5294 |
| CK | 2 | Arthropods | 5 | 1.2033 | 2.7950 | 2.3077 |
| CK | 2 | Arthropods | 2 | 0.3768 | 1.2800 | 1.1429 |
| CK | 2 | Arthropods | 8 | 1.5558 | 3.2140 | 1.9375 |
| CK | 2 | Arthropods | 6 | 1.4710 | 3.6725 | 2.6364 |
| CK | 3 | Arthropods | 6 | 1.7141 | 5.2326 | 3.7500 |
| CK | 3 | Arthropods | 9 | 1.8678 | 4.6992 | 2.5000 |
| CK | 3 | Arthropods | 4 | 0.8856 | 1.8060 | 1.3750 |
| CK | 3 | Arthropods | 4 | 0.6337 | 1.4211 | 1.2000 |
| CK | 3 | Arthropods | 7 | 1.5596 | 3.3684 | 2.0000 |
| CK | 1 | Herbivores | 4 | 1.3174 | 3.5579 | 2.8889 |
| CK | 1 | Herbivores | 4 | 1.0386 | 2.5319 | 2.1818 |
| CK | 1 | Herbivores | 3 | 0.9689 | 2.4648 | 2.1875 |
| CK | 1 | Herbivores | 5 | 1.2774 | 3.0423 | 2.2500 |
| CK | 1 | Herbivores | 6 | 1.5868 | 4.0909 | 2.5000 |
| CK | 2 | Herbivores | 5 | 0.8981 | 1.7575 | 1.3529 |
| CK | 2 | Herbivores | 3 | 0.8287 | 2.1529 | 2.0000 |
| CK | 2 | Herbivores | 1 | 0.0000 | 1.0000 | 1.0000 |
| CK | 2 | Herbivores | 4 | 1.0720 | 2.3151 | 1.6250 |
| CK | 2 | Herbivores | 4 | 0.9932 | 2.3725 | 2.0000 |
| CK | 3 | Herbivores | 4 | 1.3421 | 3.6667 | 2.7500 |
| CK | 3 | Herbivores | 3 | 0.9503 | 2.2727 | 1.6667 |
| CK | 3 | Herbivores | 3 | 1.0986 | 3.0000 | 3.0000 |
| CK | 3 | Herbivores | 1 | 0.0000 | 1.0000 | 1.0000 |
| CK | 3 | Herbivores | 2 | 0.5004 | 1.4706 | 1.2500 |
| CK | 1 | Carnivores | 1 | 0.0000 | 1.0000 | 1.0000 |
| CK | 1 | Carnivores | 1 | 0.0000 | 1.0000 | 1.0000 |
| CK | 1 | Carnivores | 1 | 0.0000 | 1.0000 | 1.0000 |
| CK | 1 | Carnivores | 5 | 1.3667 | 3.2703 | 2.2000 |
| CK | 1 | Carnivores | 3 | 0.7963 | 1.8148 | 1.4000 |
| CK | 2 | Carnivores | 0 | 0.0000 | 0.0000 | 0.0000 |
| CK | 2 | Carnivores | 1 | 0.0000 | 1.0000 | 1.0000 |
| CK | 2 | Carnivores | 1 | 0.0000 | 1.0000 | 1.0000 |
| CK | 2 | Carnivores | 2 | 0.6931 | 2.0000 | 2.0000 |
| CK | 2 | Carnivores | 1 | 0.0000 | 1.0000 | 1.0000 |
| CK | 3 | Carnivores | 1 | 0.0000 | 1.0000 | 1.0000 |
| CK | 3 | Carnivores | 2 | 0.6365 | 1.8000 | 1.5000 |
| CK | 3 | Carnivores | 0 | 0.0000 | 0.0000 | 0.0000 |
| CK | 3 | Carnivores | 1 | 0.0000 | 1.0000 | 1.0000 |
| CK | 3 | Carnivores | 3 | 1.0397 | 2.6667 | 2.0000 |
| CK | 1 | Omnivores | 0 | 0.0000 | 0.0000 | 0.0000 |
| CK | 1 | Omnivores | 1 | 0.0000 | 1.0000 | 1.0000 |
| CK | 1 | Omnivores | 0 | 0.0000 | 0.0000 | 0.0000 |
| CK | 1 | Omnivores | 0 | 0.0000 | 0.0000 | 0.0000 |
| CK | 1 | Omnivores | 1 | 0.0000 | 1.0000 | 1.0000 |
| CK | 2 | Omnivores | 2 | 0.6365 | 1.8000 | 1.5000 |
| CK | 2 | Omnivores | 1 | 0.0000 | 1.0000 | 1.0000 |
| CK | 2 | Omnivores | 0 | 0.0000 | 0.0000 | 0.0000 |
| CK | 2 | Omnivores | 2 | 0.6365 | 1.8000 | 1.5000 |
| CK | 2 | Omnivores | 1 | 0.0000 | 1.0000 | 1.0000 |
| CK | 3 | Omnivores | 1 | 0.0000 | 1.0000 | 1.0000 |
| CK | 3 | Omnivores | 4 | 1.1218 | 2.4701 | 1.7000 |
| CK | 3 | Omnivores | 1 | 0.0000 | 1.0000 | 1.0000 |
| CK | 3 | Omnivores | 2 | 0.2338 | 1.1327 | 1.0667 |
| CK | 3 | Omnivores | 2 | 0.6931 | 2.0000 | 2.0000 |
| Re_tall_Forbs | 1 | Arthropods | 7 | 1.2657 | 2.3704 | 1.6000 |
| Re_tall_Forbs | 1 | Arthropods | 6 | 1.5062 | 3.4353 | 2.0625 |
| Re_tall_Forbs | 1 | Arthropods | 6 | 1.2206 | 2.3690 | 1.6111 |
| Re_tall_Forbs | 1 | Arthropods | 12 | 2.1757 | 6.7493 | 3.5000 |
| Re_tall_Forbs | 1 | Arthropods | 8 | 1.4146 | 3.0266 | 2.0833 |
| Re_tall_Forbs | 2 | Arthropods | 6 | 1.6054 | 4.4454 | 3.2857 |
| Re_tall_Forbs | 2 | Arthropods | 11 | 2.0651 | 6.4198 | 4.1429 |
| Re_tall_Forbs | 2 | Arthropods | 11 | 1.9022 | 4.5156 | 2.4286 |
| Re_tall_Forbs | 2 | Arthropods | 11 | 1.5400 | 3.0000 | 1.9000 |
| Re_tall_Forbs | 2 | Arthropods | 13 | 1.8588 | 4.2156 | 2.3500 |
| Re_tall_Forbs | 3 | Arthropods | 12 | 1.9408 | 4.6117 | 2.4737 |
| Re_tall_Forbs | 3 | Arthropods | 10 | 1.9811 | 5.2319 | 2.7143 |
| Re_tall_Forbs | 3 | Arthropods | 8 | 1.8763 | 5.4878 | 3.7500 |
| Re_tall_Forbs | 3 | Arthropods | 5 | 1.0539 | 2.1157 | 1.5217 |
| Re_tall_Forbs | 3 | Arthropods | 10 | 1.7651 | 3.8136 | 2.1429 |
| Re_tall_Forbs | 1 | Herbivores | 5 | 0.9516 | 1.8647 | 1.4000 |
| Re_tall_Forbs | 1 | Herbivores | 4 | 1.1200 | 2.4545 | 1.6875 |
| Re_tall_Forbs | 1 | Herbivores | 5 | 0.9905 | 1.9538 | 1.4444 |
| Re_tall_Forbs | 1 | Herbivores | 9 | 1.8721 | 5.1116 | 2.9500 |
| Re_tall_Forbs | 1 | Herbivores | 5 | 1.1531 | 2.3226 | 1.6000 |
| Re_tall_Forbs | 2 | Herbivores | 4 | 1.1627 | 2.8444 | 2.2857 |
| Re_tall_Forbs | 2 | Herbivores | 6 | 1.6046 | 4.5714 | 3.4286 |
| Re_tall_Forbs | 2 | Herbivores | 6 | 1.1792 | 2.3511 | 1.6429 |
| Re_tall_Forbs | 2 | Herbivores | 9 | 1.1689 | 1.9775 | 1.4333 |
| Re_tall_Forbs | 2 | Herbivores | 10 | 1.5636 | 3.0388 | 1.8500 |
| Re_tall_Forbs | 3 | Herbivores | 7 | 1.7490 | 5.0000 | 3.7500 |
| Re_tall_Forbs | 3 | Herbivores | 4 | 1.3863 | 4.0000 | 4.0000 |
| Re_tall_Forbs | 3 | Herbivores | 5 | 1.5607 | 4.5000 | 3.0000 |
| Re_tall_Forbs | 3 | Herbivores | 0 | 0.0000 | 0.0000 | 0.0000 |
| Re_tall_Forbs | 3 | Herbivores | 3 | 1.0986 | 3.0000 | 3.0000 |
| Re_tall_Forbs | 1 | Carnivores | 2 | 0.4506 | 1.3846 | 1.2000 |
| Re_tall_Forbs | 1 | Carnivores | 2 | 0.6365 | 1.8000 | 1.5000 |
| Re_tall_Forbs | 1 | Carnivores | 1 | 0.0000 | 1.0000 | 1.0000 |
| Re_tall_Forbs | 1 | Carnivores | 3 | 1.0362 | 2.6889 | 2.2000 |
| Re_tall_Forbs | 1 | Carnivores | 1 | 0.0000 | 1.0000 | 1.0000 |
| Re_tall_Forbs | 2 | Carnivores | 1 | 0.0000 | 1.0000 | 1.0000 |
| Re_tall_Forbs | 2 | Carnivores | 3 | 1.0986 | 3.0000 | 3.0000 |
| Re_tall_Forbs | 2 | Carnivores | 2 | 0.6365 | 1.8000 | 1.5000 |
| Re_tall_Forbs | 2 | Carnivores | 1 | 0.0000 | 1.0000 | 1.0000 |
| Re_tall_Forbs | 2 | Carnivores | 1 | 0.0000 | 1.0000 | 1.0000 |
| Re_tall_Forbs | 3 | Carnivores | 3 | 1.0549 | 2.7778 | 2.5000 |
| Re_tall_Forbs | 3 | Carnivores | 3 | 1.0114 | 2.5714 | 2.0000 |
| Re_tall_Forbs | 3 | Carnivores | 1 | 0.0000 | 1.0000 | 1.0000 |
| Re_tall_Forbs | 3 | Carnivores | 1 | 0.0000 | 1.0000 | 1.0000 |
| Re_tall_Forbs | 3 | Carnivores | 3 | 1.0042 | 2.5789 | 2.3333 |
| Re_tall_Forbs | 1 | Omnivores | 0 | 0.0000 | 0.0000 | 0.0000 |
| Re_tall_Forbs | 1 | Omnivores | 0 | 0.0000 | 0.0000 | 0.0000 |
| Re_tall_Forbs | 1 | Omnivores | 0 | 0.0000 | 0.0000 | 0.0000 |
| Re_tall_Forbs | 1 | Omnivores | 0 | 0.0000 | 0.0000 | 0.0000 |
| Re_tall_Forbs | 1 | Omnivores | 2 | 0.6931 | 2.0000 | 2.0000 |
| Re_tall_Forbs | 2 | Omnivores | 1 | 0.0000 | 1.0000 | 1.0000 |
| Re_tall_Forbs | 2 | Omnivores | 2 | 0.6931 | 2.0000 | 2.0000 |
| Re_tall_Forbs | 2 | Omnivores | 3 | 0.9743 | 2.4615 | 2.0000 |
| Re_tall_Forbs | 2 | Omnivores | 1 | 0.0000 | 1.0000 | 1.0000 |
| Re_tall_Forbs | 2 | Omnivores | 2 | 0.3365 | 1.2321 | 1.1176 |
| Re_tall_Forbs | 3 | Omnivores | 2 | 0.6077 | 1.7153 | 1.4211 |
| Re_tall_Forbs | 3 | Omnivores | 3 | 0.6837 | 1.5882 | 1.2857 |
| Re_tall_Forbs | 3 | Omnivores | 2 | 0.5004 | 1.4706 | 1.2500 |
| Re_tall_Forbs | 3 | Omnivores | 4 | 0.7191 | 1.5488 | 1.2609 |
| Re_tall_Forbs | 3 | Omnivores | 4 | 0.8711 | 1.8692 | 1.4286 |
| Re_short_Forbs | 1 | Arthropods | 13 | 1.9705 | 4.2642 | 2.2308 |
| Re_short_Forbs | 1 | Arthropods | 4 | 1.0998 | 2.4100 | 1.6774 |
| Re_short_Forbs | 1 | Arthropods | 10 | 1.8477 | 4.4720 | 2.4286 |
| Re_short_Forbs | 1 | Arthropods | 7 | 1.5183 | 3.1977 | 1.9333 |
| Re_short_Forbs | 1 | Arthropods | 7 | 1.3252 | 2.5491 | 1.6757 |
| Re_short_Forbs | 2 | Arthropods | 11 | 1.3754 | 2.4516 | 1.6364 |
| Re_short_Forbs | 2 | Arthropods | 11 | 1.9702 | 5.5764 | 3.7000 |
| Re_short_Forbs | 2 | Arthropods | 15 | 1.7385 | 2.8790 | 1.7400 |
| Re_short_Forbs | 2 | Arthropods | 8 | 1.5141 | 2.9002 | 1.7857 |
| Re_short_Forbs | 2 | Arthropods | 8 | 1.0845 | 1.8245 | 1.3654 |
| Re_short_Forbs | 3 | Arthropods | 7 | 1.4356 | 3.2609 | 2.5000 |
| Re_short_Forbs | 3 | Arthropods | 11 | 1.7108 | 3.2291 | 1.8889 |
| Re_short_Forbs | 3 | Arthropods | 10 | 1.3707 | 2.3164 | 1.5593 |
| Re_short_Forbs | 3 | Arthropods | 11 | 2.1974 | 7.5625 | 4.4000 |
| Re_short_Forbs | 3 | Arthropods | 8 | 1.4075 | 2.6076 | 1.6818 |
| Re_short_Forbs | 1 | Herbivores | 7 | 1.8019 | 5.4468 | 4.0000 |
| Re_short_Forbs | 1 | Herbivores | 2 | 0.6365 | 1.8000 | 1.5000 |
| Re_short_Forbs | 1 | Herbivores | 5 | 1.4862 | 4.0000 | 3.1111 |
| Re_short_Forbs | 1 | Herbivores | 3 | 1.0397 | 2.6667 | 2.0000 |
| Re_short_Forbs | 1 | Herbivores | 4 | 1.2566 | 3.1947 | 2.3750 |
| Re_short_Forbs | 2 | Herbivores | 6 | 1.4209 | 3.3088 | 2.1429 |
| Re_short_Forbs | 2 | Herbivores | 7 | 1.6045 | 3.9555 | 2.4500 |
| Re_short_Forbs | 2 | Herbivores | 10 | 2.1405 | 7.5385 | 4.6667 |
| Re_short_Forbs | 2 | Herbivores | 5 | 1.5438 | 4.4138 | 3.2000 |
| Re_short_Forbs | 2 | Herbivores | 4 | 1.3322 | 3.5957 | 2.6000 |
| Re_short_Forbs | 3 | Herbivores | 3 | 1.0549 | 2.7778 | 2.5000 |
| Re_short_Forbs | 3 | Herbivores | 5 | 1.4708 | 3.8462 | 2.5000 |
| Re_short_Forbs | 3 | Herbivores | 5 | 1.5089 | 4.1538 | 3.0000 |
| Re_short_Forbs | 3 | Herbivores | 7 | 1.7095 | 4.5918 | 3.0000 |
| Re_short_Forbs | 3 | Herbivores | 4 | 0.6551 | 1.4727 | 1.2273 |
| Re_short_Forbs | 1 | Carnivores | 5 | 1.4615 | 3.8788 | 2.6667 |
| Re_short_Forbs | 1 | Carnivores | 1 | 0.0000 | 1.0000 | 1.0000 |
| Re_short_Forbs | 1 | Carnivores | 3 | 0.9165 | 2.2830 | 1.8333 |
| Re_short_Forbs | 1 | Carnivores | 2 | 0.6730 | 1.9231 | 1.6667 |
| Re_short_Forbs | 1 | Carnivores | 2 | 0.4506 | 1.3846 | 1.2000 |
| Re_short_Forbs | 2 | Carnivores | 3 | 1.0986 | 3.0000 | 3.0000 |
| Re_short_Forbs | 2 | Carnivores | 2 | 0.6730 | 1.9231 | 1.6667 |
| Re_short_Forbs | 2 | Carnivores | 2 | 0.6365 | 1.8000 | 1.5000 |
| Re_short_Forbs | 2 | Carnivores | 1 | 0.0000 | 1.0000 | 1.0000 |
| Re_short_Forbs | 2 | Carnivores | 1 | 0.0000 | 1.0000 | 1.0000 |
| Re_short_Forbs | 3 | Carnivores | 1 | 0.0000 | 1.0000 | 1.0000 |
| Re_short_Forbs | 3 | Carnivores | 5 | 1.5607 | 4.5000 | 3.0000 |
| Re_short_Forbs | 3 | Carnivores | 3 | 1.0114 | 2.5714 | 2.0000 |
| Re_short_Forbs | 3 | Carnivores | 3 | 1.0397 | 2.6667 | 2.0000 |
| Re_short_Forbs | 3 | Carnivores | 2 | 0.5623 | 1.6000 | 1.3333 |
| Re_short_Forbs | 1 | Omnivores | 1 | 0.0000 | 1.0000 | 1.0000 |
| Re_short_Forbs | 1 | Omnivores | 1 | 0.0000 | 1.0000 | 1.0000 |
| Re_short_Forbs | 1 | Omnivores | 2 | 0.1500 | 1.0713 | 1.0357 |
| Re_short_Forbs | 1 | Omnivores | 2 | 0.2338 | 1.1327 | 1.0667 |
| Re_short_Forbs | 1 | Omnivores | 1 | 0.0000 | 1.0000 | 1.0000 |
| Re_short_Forbs | 2 | Omnivores | 2 | 0.1520 | 1.0726 | 1.0364 |
| Re_short_Forbs | 2 | Omnivores | 2 | 0.1985 | 1.1050 | 1.0526 |
| Re_short_Forbs | 2 | Omnivores | 3 | 0.4087 | 1.2444 | 1.1200 |
| Re_short_Forbs | 2 | Omnivores | 2 | 0.3768 | 1.2800 | 1.1429 |
| Re_short_Forbs | 2 | Omnivores | 3 | 0.3068 | 1.1563 | 1.0769 |
| Re_short_Forbs | 3 | Omnivores | 3 | 0.5360 | 1.3740 | 1.1818 |
| Re_short_Forbs | 3 | Omnivores | 1 | 0.0000 | 1.0000 | 1.0000 |
| Re_short_Forbs | 3 | Omnivores | 2 | 0.3908 | 1.2981 | 1.1525 |
| Re_short_Forbs | 3 | Omnivores | 1 | 0.0000 | 1.0000 | 1.0000 |
| Re_short_Forbs | 3 | Omnivores | 2 | 0.6365 | 1.8000 | 1.5000 |
| Re_tall_Grasses_Sedges | 1 | Arthropods | 3 | 0.7508 | 2.0117 | 1.7500 |
| Re_tall_Grasses_Sedges | 1 | Arthropods | 6 | 1.3774 | 3.2377 | 2.5333 |
| Re_tall_Grasses_Sedges | 1 | Arthropods | 6 | 1.1637 | 2.1978 | 1.5385 |
| Re_tall_Grasses_Sedges | 1 | Arthropods | 10 | 1.6857 | 3.4105 | 2.0000 |
| Re_tall_Grasses_Sedges | 1 | Arthropods | 5 | 0.9872 | 2.1886 | 1.6739 |
| Re_tall_Grasses_Sedges | 2 | Arthropods | 10 | 1.6242 | 3.6464 | 2.4595 |
| Re_tall_Grasses_Sedges | 2 | Arthropods | 9 | 1.6088 | 3.3158 | 2.0000 |
| Re_tall_Grasses_Sedges | 2 | Arthropods | 7 | 1.5177 | 3.4490 | 2.1667 |
| Re_tall_Grasses_Sedges | 2 | Arthropods | 9 | 1.8881 | 5.2514 | 3.1000 |
| Re_tall_Grasses_Sedges | 2 | Arthropods | 6 | 0.9872 | 2.2739 | 1.8772 |
| Re_tall_Grasses_Sedges | 3 | Arthropods | 7 | 1.0690 | 1.9053 | 1.4048 |
| Re_tall_Grasses_Sedges | 3 | Arthropods | 6 | 0.9049 | 1.6667 | 1.3043 |
| Re_tall_Grasses_Sedges | 3 | Arthropods | 6 | 1.5119 | 3.7532 | 2.4286 |
| Re_tall_Grasses_Sedges | 3 | Arthropods | 9 | 1.3161 | 2.4627 | 1.6786 |
| Re_tall_Grasses_Sedges | 3 | Arthropods | 7 | 0.9204 | 1.7082 | 1.3276 |
| Re_tall_Grasses_Sedges | 1 | Herbivores | 1 | 0.0000 | 1.0000 | 1.0000 |
| Re_tall_Grasses_Sedges | 1 | Herbivores | 3 | 0.8018 | 1.8519 | 1.4286 |
| Re_tall_Grasses_Sedges | 1 | Herbivores | 2 | 0.2573 | 1.1529 | 1.0769 |
| Re_tall_Grasses_Sedges | 1 | Herbivores | 7 | 1.7141 | 4.4118 | 2.5000 |
| Re_tall_Grasses_Sedges | 1 | Herbivores | 2 | 0.1732 | 1.0868 | 1.0435 |
| Re_tall_Grasses_Sedges | 2 | Herbivores | 8 | 1.4654 | 2.9127 | 1.8214 |
| Re_tall_Grasses_Sedges | 2 | Herbivores | 6 | 1.2835 | 2.6276 | 1.7619 |
| Re_tall_Grasses_Sedges | 2 | Herbivores | 6 | 1.4088 | 3.2051 | 2.0833 |
| Re_tall_Grasses_Sedges | 2 | Herbivores | 6 | 1.5320 | 3.6667 | 2.2000 |
| Re_tall_Grasses_Sedges | 2 | Herbivores | 6 | 0.9872 | 2.2739 | 1.8772 |
| Re_tall_Grasses_Sedges | 3 | Herbivores | 4 | 1.3398 | 3.6885 | 3.0000 |
| Re_tall_Grasses_Sedges | 3 | Herbivores | 2 | 0.6931 | 2.0000 | 2.0000 |
| Re_tall_Grasses_Sedges | 3 | Herbivores | 3 | 0.9743 | 2.4615 | 2.0000 |
| Re_tall_Grasses_Sedges | 3 | Herbivores | 6 | 1.3157 | 2.6036 | 1.7000 |
| Re_tall_Grasses_Sedges | 3 | Herbivores | 4 | 1.1807 | 2.7524 | 1.8889 |
| Re_tall_Grasses_Sedges | 1 | Carnivores | 1 | 0.0000 | 1.0000 | 1.0000 |
| Re_tall_Grasses_Sedges | 1 | Carnivores | 2 | 0.6365 | 1.8000 | 1.5000 |
| Re_tall_Grasses_Sedges | 1 | Carnivores | 2 | 0.6931 | 2.0000 | 2.0000 |
| Re_tall_Grasses_Sedges | 1 | Carnivores | 2 | 0.6365 | 1.8000 | 1.5000 |
| Re_tall_Grasses_Sedges | 1 | Carnivores | 2 | 0.6730 | 1.9231 | 1.6667 |
| Re_tall_Grasses_Sedges | 2 | Carnivores | 0 | 0.0000 | 0.0000 | 0.0000 |
| Re_tall_Grasses_Sedges | 2 | Carnivores | 1 | 0.0000 | 1.0000 | 1.0000 |
| Re_tall_Grasses_Sedges | 2 | Carnivores | 1 | 0.0000 | 1.0000 | 1.0000 |
| Re_tall_Grasses_Sedges | 2 | Carnivores | 1 | 0.0000 | 1.0000 | 1.0000 |
| Re_tall_Grasses_Sedges | 2 | Carnivores | 0 | 0.0000 | 0.0000 | 0.0000 |
| Re_tall_Grasses_Sedges | 3 | Carnivores | 1 | 0.0000 | 1.0000 | 1.0000 |
| Re_tall_Grasses_Sedges | 3 | Carnivores | 2 | 0.6931 | 2.0000 | 2.0000 |
| Re_tall_Grasses_Sedges | 3 | Carnivores | 1 | 0.0000 | 1.0000 | 1.0000 |
| Re_tall_Grasses_Sedges | 3 | Carnivores | 0 | 0.0000 | 0.0000 | 0.0000 |
| Re_tall_Grasses_Sedges | 3 | Carnivores | 1 | 0.0000 | 1.0000 | 1.0000 |
| Re_tall_Grasses_Sedges | 1 | Omnivores | 1 | 0.0000 | 1.0000 | 1.0000 |
| Re_tall_Grasses_Sedges | 1 | Omnivores | 1 | 0.0000 | 1.0000 | 1.0000 |
| Re_tall_Grasses_Sedges | 1 | Omnivores | 2 | 0.5623 | 1.6000 | 1.3333 |
| Re_tall_Grasses_Sedges | 1 | Omnivores | 1 | 0.0000 | 1.0000 | 1.0000 |
| Re_tall_Grasses_Sedges | 1 | Omnivores | 1 | 0.0000 | 1.0000 | 1.0000 |
| Re_tall_Grasses_Sedges | 2 | Omnivores | 2 | 0.2664 | 1.1611 | 1.0811 |
| Re_tall_Grasses_Sedges | 2 | Omnivores | 2 | 0.5623 | 1.6000 | 1.3333 |
| Re_tall_Grasses_Sedges | 2 | Omnivores | 0 | 0.0000 | 0.0000 | 0.0000 |
| Re_tall_Grasses_Sedges | 2 | Omnivores | 2 | 0.3768 | 1.2800 | 1.1429 |
| Re_tall_Grasses_Sedges | 2 | Omnivores | 0 | 0.0000 | 0.0000 | 0.0000 |
| Re_tall_Grasses_Sedges | 3 | Omnivores | 2 | 0.1105 | 1.0476 | 1.0238 |
| Re_tall_Grasses_Sedges | 3 | Omnivores | 2 | 0.1732 | 1.0868 | 1.0435 |
| Re_tall_Grasses_Sedges | 3 | Omnivores | 2 | 0.3768 | 1.2800 | 1.1429 |
| Re_tall_Grasses_Sedges | 3 | Omnivores | 3 | 0.2911 | 1.1450 | 1.0714 |
| Re_tall_Grasses_Sedges | 3 | Omnivores | 2 | 0.0859 | 1.0345 | 1.0172 |

## Supplementary Figures

**
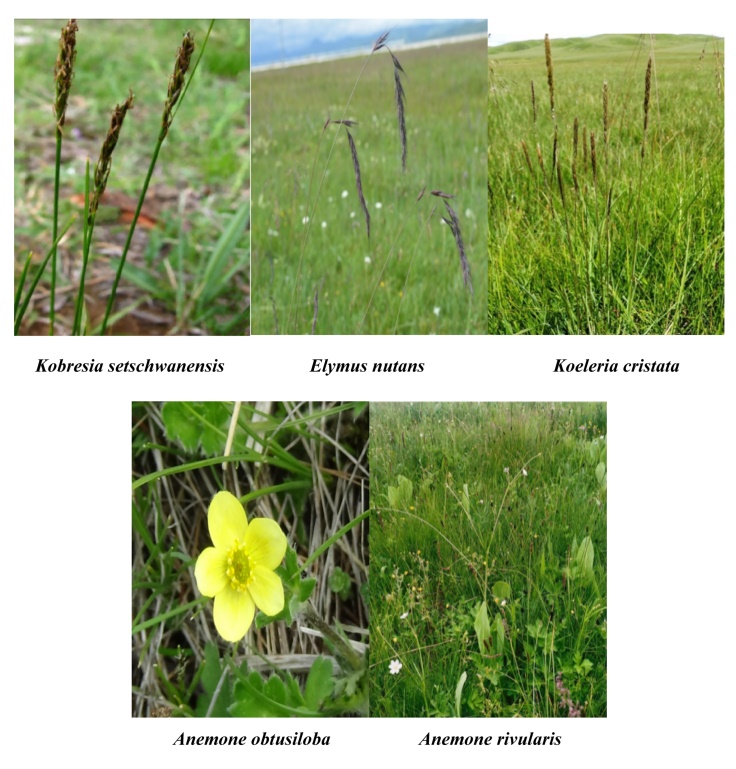
**

Figure S1. Typical vegetation on the Eastern Qinghai-Tibet Plateau meadow


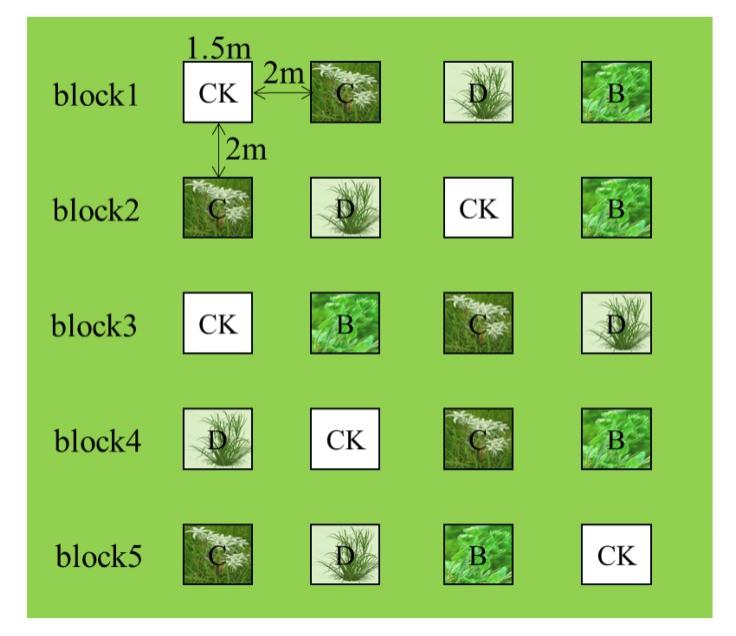


Figure S2. Experiment photos showing all treatments and plots


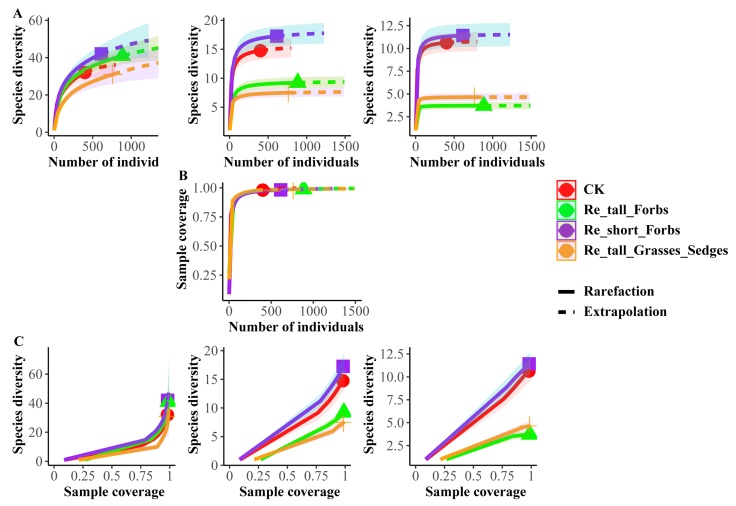


Figure S3. (A) Sample-size-based and (C) coverage-based rarefaction (solid line segment) and extrapolation (dotted line segments) sampling curves with 95% confidence intervals (shaded areas) for the arthropods data of four treatments, separately by diversity order: species richness (*q* = 0, left panel), exponential of Shannon entropy (*q* = 1, middle panel) and inverse Simpson concentration (*q* = 2, right panel). The solid dots/triangles represent the reference samples. (B) Sample completeness curves linking curves in (A) and (C).


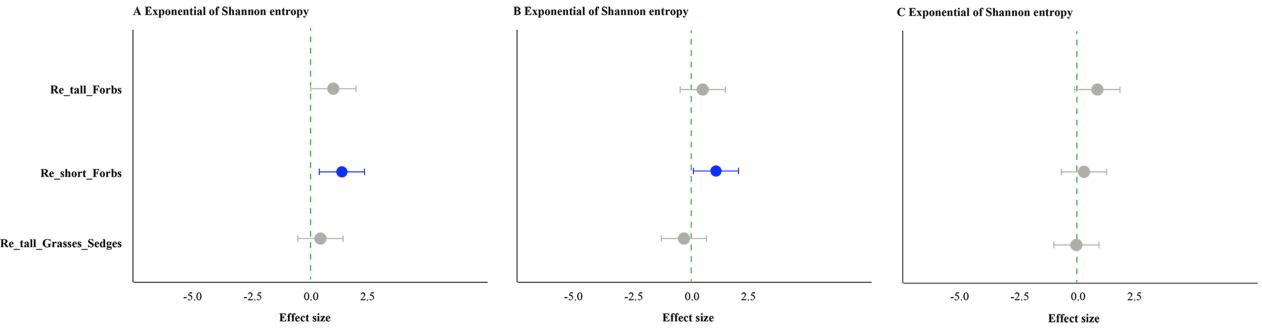


Figure S4. The effect of plant species removal on the exponential of Shannon entropy of arthropod functional groups, as determined by linear mixed-effects models (LMMs). Herbivores (A), carnivores (B), and omnivores (C). The comments in the figure correspond to those shown in Figure 1.


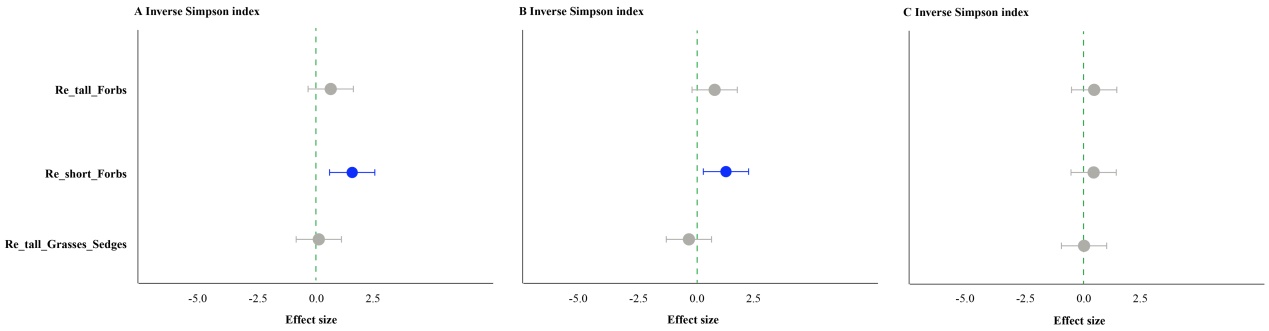


Figure S5. The effect of plant species removal on the inverse Simpson index of arthropod functional groups, as determined by linear mixed-effects models (LMMs). Herbivores (A), carnivores (B), and omnivores (C). The comments in the figure correspond to those shown in Figure 1.


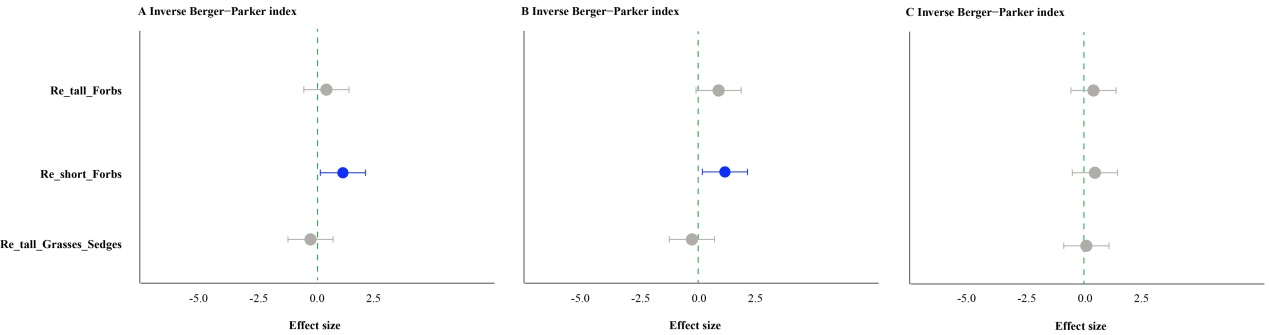


Figure S6. The effect of plant species removal on the inverse Berger-Parker index of arthropod functional groups, as determined by linear mixed-effects models (LMMs). Herbivores (A), carnivores (B), and omnivores (C). The comments in the figure correspond to those shown in Figure 1.


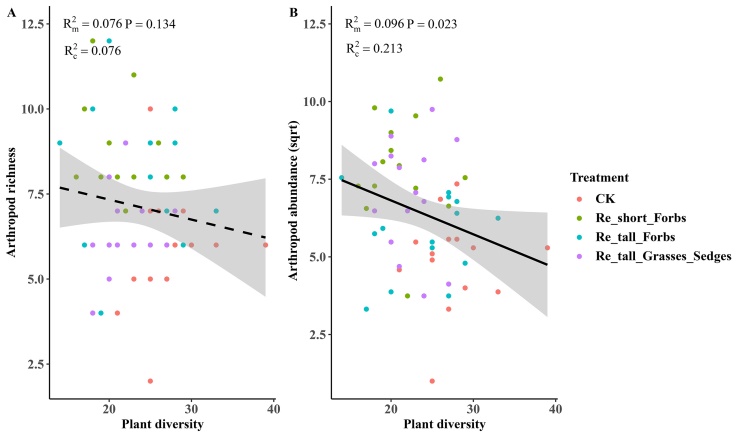


Figure S7. Relationship between plant diversity and arthropod diversity and abundance. The figure shows the plant diversity and arthropod richness (A), plant diversity and arthropod abundance (B). The figure includes the R^2^ value and P value of the regression model, along with a 95% confidence interval.


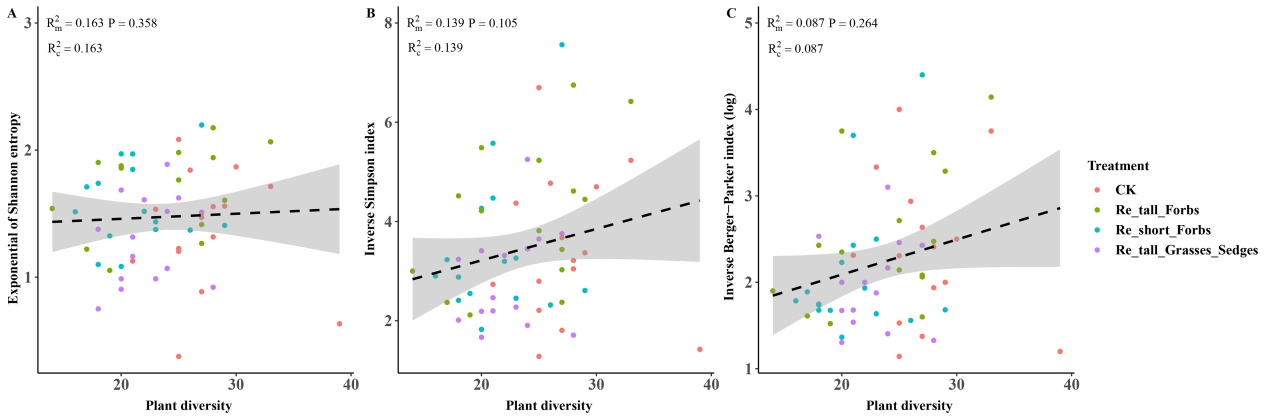


Figure S8. Relationship between plant diversity and arthropod diversity. The figure shows the plant diversity and exponential of Shannon entropy of arthropod (A), plant diversity and arthropod inverse Simpson index (B) and plant diversity and arthropod inverse Berger-parker index (C). The figure includes the R^2^ value and P value of the regression model, along with a 95% confidence interval.


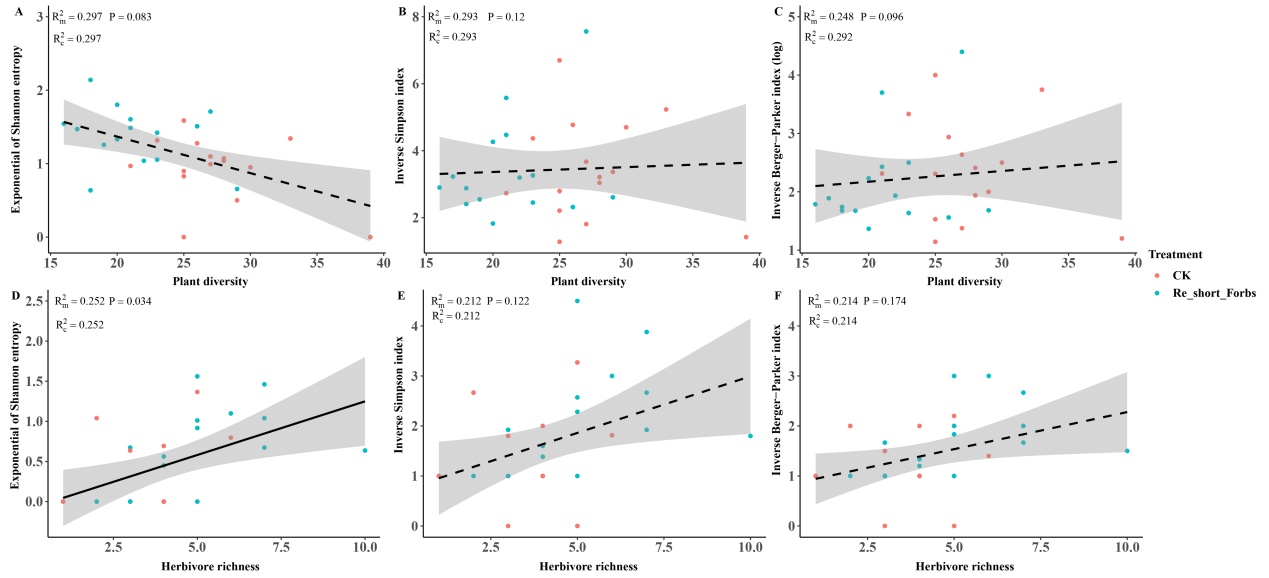


Figure S9. The relationships between plant diversity and arthropod trophic groups in the removal of short forbs and the control. The figure shows the plant diversity and exponential of Shannon entropy of herbivore (A), plant diversity and herbivore inverse Simpson index (B), plant diversity and herbivore inverse Berger-parker index (C), herbivore richness and exponential of Shannon entropy of carnivore (D), herbivore richness and carnivore inverse Simpson index (E), herbivore richness and carnivore inverse Berger-Parker index (F). The figure includes the R^2^ value and P value of the regression model, along with a 95% confidence interval.


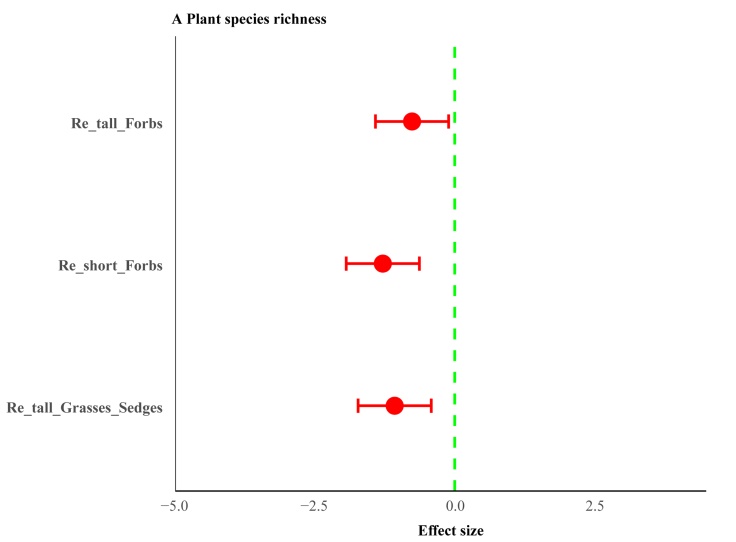


Figure S10. Quantifying the effects of plant species remove on plant communities. In the linear mixing model, the positive (blue) or negative (red) effect values of different species removal on plant species richness was expressed by the estimated values. The comments in the figure correspond to those shown in Figure 1.
